# The lung tissue microbiota of mild and moderate chronic obstructive pulmonary disease

**DOI:** 10.1186/s40168-017-0381-4

**Published:** 2018-01-09

**Authors:** Alexa A. Pragman, Tianmeng Lyu, Joshua A. Baller, Trevor J. Gould, Rosemary F. Kelly, Cavan S. Reilly, Richard E. Isaacson, Chris H. Wendt

**Affiliations:** 1 Department of Medicine, University of Minnesota and Minneapolis Veterans Affairs Medical Center, Minneapolis VA Health Care System, Research Service (151), 1 Veterans Drive, Minneapolis, MN 55417 USA; 2 Division of Biostatistics, University of Minnesota School of Public Health, MMC 303 Mayo, 8303A, 420 Delaware St. SE, Minneapolis, MN 55455 USA; 3 Minnesota Supercomputing Institute, University of Minnesota, Room 599 Walter Library, 3721A, 117 Pleasant St. SE, Minneapolis, MN 55455 USA; 4 Biological Science Dean’s Office, University of Minnesota Informatics Institute, Room 123 SnH, 6174A, 1475 Gortner Ave., St. Paul, MN 55108 USA; 5 Division of Cardiothoracic Surgery, University of Minnesota and Minneapolis Veterans Affairs Medical Center, 1 Veterans Dr., Minneapolis, MN 55417 USA; 6 Department of Veterinary and Biomedical Sciences, University of Minnesota, Room 205G VetS, 6187A, 1971 Commonwealth Ave., St. Paul, MN 55108 USA

**Keywords:** RNA, Ribosomal, 16S, Emigration and immigration, Lung, Pulmonary disease, chronic obstructive, Microbiota, Streptococcus

## Abstract

**Background:**

Oral taxa are often found in the chronic obstructive pulmonary disease (COPD) lung microbiota, but it is not clear if this is due to a physiologic process such as aspiration or experimental contamination at the time of specimen collection.

**Methods:**

Microbiota samples were obtained from nine subjects with mild or moderate COPD by swabbing lung tissue and upper airway sites during lung lobectomy. Lung specimens were not contaminated with upper airway taxa since they were obtained surgically. The microbiota were analyzed with 16S rRNA gene qPCR and 16S rRNA gene hypervariable region 3 (V3) sequencing. Data analyses were performed using QIIME, SourceTracker, and R.

**Results:**

*Streptococcus* was the most common genus in the oral, bronchial, and lung tissue samples, and multiple other taxa were present in both the upper and lower airways. Each subject’s own bronchial and lung tissue microbiota were more similar to each other than were the bronchial and lung tissue microbiota of two different subjects (permutation test, *p* = 0.0139), indicating more within-subject similarity than between-subject similarity at these two lung sites. Principal coordinate analysis of all subject samples revealed clustering by anatomic sampling site (PERMANOVA, *p* = 0.001), but not by subject. SourceTracker analysis found that the sources of the lung tissue microbiota were 21.1% (mean) oral microbiota, 8.7% nasal microbiota, and 70.1% unknown. An analysis using the neutral theory of community ecology revealed that the lung tissue microbiota closely reflects the bronchial, oral, and nasal microbiota (immigration parameter estimates 0.69, 0.62, and 0.74, respectively), with some evidence of ecologic drift occurring in the lung tissue.

**Conclusion:**

This is the first study to evaluate the mild-moderate COPD lung tissue microbiota without potential for upper airway contamination of the lung samples. In our small study of subjects with COPD, we found oral and nasal bacteria in the lung tissue microbiota, confirming that aspiration is a source of the COPD lung microbiota.

## Background

High-throughput sequencing techniques have revolutionized lung microbiota studies, leading to the realization that healthy lungs are not sterile; rather, they harbor complex microbiota. Several studies of the microbiota of healthy and chronic obstructive pulmonary disease (COPD)-affected lungs from bronchoalveolar lavage (BAL) or sputum have been described using molecular methods [1–13]. These studies utilized samples obtained through the upper airway, such as induced sputum, bronchoalveolar lavage, and endotracheal aspirate. Many of these studies identified oral bacteria in their lower airway lung samples. Charlson et al. analyzed BAL fluid from healthy volunteers using a 2-scope technique and compared the lung microbiota to the nasal and oral microbiota [2]. The lung microbiota was nearly indistinguishable from the oral microbiota, but the authors were unable to determine if their findings resulted from aspiration vs. contamination of the bronchoscope during insertion through the mouth.

Our study of the lung microbiota in COPD also found oral taxa in the lung [1]. Our analysis of BAL fluid from 22 patients with moderate or severe COPD and 10 healthy patients identified a greater proportion of oral bacteria (such as *Desulfobulbus*, *Abiotrophia*, and *Selenomonas*) in the COPD microbiota than in the healthy lung microbiota. Several subsequent studies of the lung microbiota using BAL had similar findings [11–14]. These studies support the hypothesis that oral bacteria found in the lung microbiota are most likely the result of aspiration of oral secretions, rather than oral contamination of the lung sample during bronchoscopy.

Aspiration of oral bacteria is the most likely source of the lung microbiota as the mouth and lungs are in direct continuity and the mouth is microbe-rich. COPD patients are prone to aspiration because of reduced laryngotracheal mechanosensitivity [15, 16] and poor coordination of breathing and swallowing [17, 18]. Aspiration has significant consequences for COPD patients due to decreased airway clearance as a result of impaired mucociliary function [19]. Accordingly, COPD patients likely both aspirate more frequently than healthy patients and fail to clear the aspirate, thereby exposing their lungs to more oral bacteria. The observed lung microbiota may be the result of rare seeding of oral bacteria followed by proliferation of oral bacteria in the lung (a process akin to “ecologic drift”), or the lung microbiota may be maintained by repeated aspiration, with little or no growth of oral bacteria in the lung. This latter process is described by the adapted island model of lung biogeography, as proposed by Dickson et al. [20]. However, no empiric studies of the COPD lung microbiota have studied ecologic drift or the adapted island model using samples obtained without potential oral contamination.

Two studies have evaluated the lung tissue microbiota in end-stage COPD at the time of lung transplantation [3, 21]. In both cases, the subjects’ end-stage COPD (with anatomic abnormalities, bronchiectasis, and prior exposure to antibiotics and corticosteroids) may have influenced the results and may not be representative of the findings in earlier-stage COPD. Notably, neither study evaluated the relatedness of upper airway and lung samples, precluding evidence concerning the aspiration of oral microbes. Therefore, the true content of the early-stage COPD lung microbiota and the potential role of aspiration remains unknown.

The rationale for the present study is that evaluation of the pathogenic character of the COPD lung microbiota has been hindered by concerns that oral taxa found in the COPD lung microbiota are the result of sample contamination rather than aspiration. Therefore, we have designed the present study to sample the mild or moderate COPD lung tissue microbiota surgically—without passing the sample through the oropharynx—and avoiding potential upper airway contamination of lung samples. Demonstrating that oral microbes are true components of the COPD lung microbiota will implicate aspiration as a potential pathogenic mechanism in COPD. We hypothesized that oral bacteria are true members of the early-stage COPD lung microbiota and exhibit ecologic drift. Some of the results of this study have been presented in the form of abstracts.

## Methods

### Subjects

Patients with COPD undergoing clinically indicated lung lobectomy for suspected or confirmed lung cancer at the Minneapolis Veterans Affairs Medical Center (MVAMC; eight subjects) or University of Minnesota Medical Center (UMMC; one subject) were offered inclusion in our study. Seven subjects were male and two were female. Inclusion criteria were as follows: (1) undergoing clinically indicated lung lobectomy, (2) age ≥ 40, (3) diagnosis of COPD by GOLD criteria (FEV_1_/FVC < 70%) [22], and (4) at least a 10 pack-year history of smoking. Exclusion criteria were as follows (1) use of antibiotics or oral corticosteroids within the last 2 months, (2) history of asthma, (3) endobronchial lesion and/or lobar atelectasis noted on imaging or during surgery, or (4) aspiration observed during intubation. Clinical data obtained via interview and chart review included gender, age, COPD severity, tobacco exposure, use of COPD medications, and recent exposure to oral corticosteroids or antibiotics.

### Sample acquisition

All subjects underwent wedge resection or intra-operative biopsy of their lung lesions for frozen section pathologic analysis to confirm lung malignancy prior to lobectomy. Only patients with confirmed malignancy and who underwent lobectomy were included in our study. All samples were obtained by swabbing lung or upper airway sites in the operating room. Following removal, the affected lobe was placed in a sterile basin for the study investigators (C.H.W. or A.A.P.) to sample the main bronchial airway and the alveolar surface of healthy-appearing peripheral lung tissue using sterile technique and nylon-flocked swabs (Copan Diagnostics, Inc., Murrieta, CA). The sutures on the main bronchial airway supplying the removed lobe were cut open and the airway was sampled consecutively with two swabs. A second scissors was used to cut into the distal healthy appearing lung parenchyma (alveolar tissue), and the interior lung surfaces were swabbed with two different swabs consecutively (note that the tumor and an adjacent margin of healthy tissue had already been removed via wedge resection). Upper airway samples were then obtained by passing a swab into the intubated subject’s oropharynx and sampling the saliva, tongue, and buccal mucosa (but not specifically sampling tonsil or teeth). A second swab was passed into the subject’s nose, sampling the anterior nares and posterior nasopharynx. Swabs were placed in sterile, DNA-free tubes and frozen at − 80 °C until DNA extraction. When two swabs were obtained from the same site, the swabs were pooled for DNA extraction and analysis. Swabs were used to provide direct samples from specific lung regions and to ensure that identical sampling techniques were used to obtain upper and lower airway samples. DNA contamination of reagents and equipment was evaluated using six negative controls consisting of unused swabs extracted, sequenced, and analyzed alongside the experimental samples.

### DNA extraction and 16S rRNA gene sequencing

DNA was extracted from the swabs using chemical and mechanical lysis as we have previously reported [1]. 16S rRNA gene hypervariable region 3 (V3) amplicons were generated via PCR amplification using primers as reported by Bartram [23]. Amplicons were sequenced on the Illumina MiSeq instrument using paired-end reads at the University of Minnesota Genomics Center. Environmental and reagent control samples consisting of unused nylon-flocked swabs were processed alongside subject samples. Samples were PCR amplified using the minimum number of PCR cycles (≤ 35) necessary to produce a visible band upon agarose gel electrophoresis. In all cases, control samples did not produce a visible band on agarose gel electrophoresis.

### Quantitative PCR

To determine 16S rRNA gene copy numbers for each sample, quantitative PCR (qPCR) was performed on 36 samples done in triplicate in 20 μl reactions using 16S rRNA qPCR primers 338-F (5′ -ACTCCTACGGGAGGCAGCAG-3′) and 518-R (5′-ATTACCGCGGCTGCTGG-3′) at a final concentration of 0.3 μmol/L for each primer. The SYBR Select Master Mix kit (Life Technologies, Grand Island, NY) was utilized for qPCR on the Stratagene (Agilent) Mx3000P ThermoCycler. Cycling conditions were performed according to the kit’s specifications (95 °C for 1 min, 55 °C for 30 s, and 95 °C for 30 s), followed by a melting curve. The standard curves for absolute quantification of 16S rRNA gene copy numbers were constructed using the DH5α *Escherichia coli* strain by initially creating an end-point PCR product of the DH5α strain with universal 16S rRNA gene primers Bact-27F and Bact-1492R [24]. The standard curve was created using tenfold serial dilutions from concentrations of 3.99 × 10^1^ to 3.99 × 10^7^ copies per milliliter.

### Data processing

**Fig. 1:**
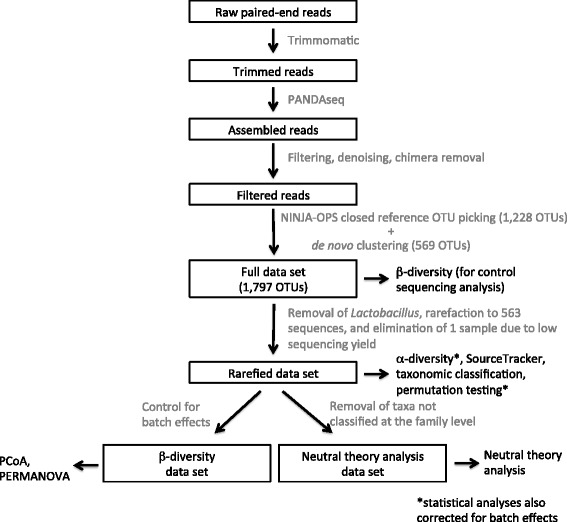
Data analysis flow chart. DNA sequences were processed into data sets using the software tools and procedures as described in the text and illustrated here as a pipeline. Boxed black text indicates significant steps in the pipeline or data sets produced for specific analyses. Gray text specifies the software tools or procedures used in the pipeline. Unboxed black text specifies the analyses performed on the indicated data sets

Paired-end reads were trimmed using trimmomatic [25], assembled together using PANDAseq [26], and the resulting sequences were subjected to filtering, denoising, and chimera removal within QIIME [27]. OTU picking at 97% identity was accomplished with a combination of ninja_ops [28] closed-reference OTU picking utilizing Greengenes (version 13_8), followed by de novo clustering of unmapped reads. The full data set underwent β-diversity analysis with Bray-Curtis distance to visualize control and sample similarities and determine subsampling depth. Control samples had low sequencing yields and did not cluster with the low abundance bronchial and peripheral lung samples. We compared the most abundant taxa in the control samples to the taxa in the subject samples. Only one taxon (*Lactobacillus*) was present in the negative control samples and present at > 1% relative abundance in the subject samples. This taxon was therefore eliminated from all subject samples. Subsampling to 563 sequences eliminated 5 of 6 negative control samples and 1 of 36 patient samples (Subject 8, Peripheral Lung) below this threshold. This rarefied data set underwent α-diversity calculations, SourceTracker analysis, taxonomic classification using Greengenes (13_8), and permutation testing. After controlling for sequencing batch effects, the β-diversity dataset (using Bray-Curtis distance) was used for principal coordinate analysis (PCoA) and PERMOVOVA. The neutral theory analysis dataset was obtained by removing from the rarefied data set taxa that were not classified at the family level. A data processing flow chart is provided in Fig. 1.

### SourceTracker

Using the rarefied data set, we employed SourceTracker [29, 30] to assess the contributions to the lung microbiota. We evaluated each subject’s bronchial and lung tissue microbiota separately and provided only the subject’s own oral and nasal microbiota as potential sources.

### Multidimensional scaling

Control and sample similarity was assessed with β-diversity using Bray-Curtis distance on the full data set. Following removal of *Lactobacillus* and rarefaction to 563 sequences, the remaining 35 subject samples were assessed again using β-diversity and PERMANOVA. PERMANOVA revealed clustering by sequencing batch, necessitating statistical adjustment for batch effects prior to further PCoA or PERMANOVA testing. Adjustment for the sequencing batch effects was accomplished within R (using biom, base stats, and vegan packages) by applying a linear model including the batch effect to the rarefied data set, resulting in the β-diversity data set. The top 10 OTUs were also plotted to indicate their contributions to the PCoA clustering.

### Permutation testing

The similarity of each subject’s paired bronchial and lung tissue samples (in comparison to the similarity between one subject’s bronchial sample and a different subject’s lung tissue sample) was assessed with permutation testing. The observed value was the average distance of the diagonal values in the Bray-Curtis distance matrix (the within-subject bronchial and lung tissue similarities). The data in each sequencing batch were then permuted separately to account for batch effects. In the first batch with three subjects, there were six permutations; in the second batch with five subjects, there were 120 permutations. In total, there were 6 × 120 = 720 permutations. The average distance was calculated for each permuted pair; then, the *p* value was calculated by the percentage that the observed value was greater than or equal to the permutation values.

### Neutral theory analysis

To prepare the full data set of 1797 OTUs for analysis, we first disposed of the 223 OTUs that were unclassified at the family level. The resulting data set had 1574 OTUs that were assigned to 286 genera. To estimate model parameters in the neutral theory model, we used the method of moments first described by Sloan [31], which uses the result from the Wright Fisher model that the equilibrium distribution for the Markov chain described by this model converges to a beta distribution. The first shape parameter of the beta distribution is the product of three factors: the average total number of reads in the lung, the immigration probability, and the average relative abundance of each microbe in one of the source sites (oral, nasal, and bronchial). The second shape parameter is similarly defined except that 1 minus the average relative abundance of each microbe in one of the source sites is used as the third factor. We defined the detection probability in the lung as the probability that the relative abundance of a microbe is greater than the detection limit which was estimated by 1 over the average total number of reads in the lung. Since the relative abundance of a microbe in the lung follows a beta distribution, the theoretical value of the detection probability can be calculated by the integration of the beta distribution from the detection limit to 1. Then, the immigration probability can be estimated by minimizing the sum of the squared differences between the theoretical values of the detection probabilities and the frequencies that the microbes are observed across the subjects. The confidence bounds around the expected values of the detection probabilities were the 95% binomial confidence intervals. All analyses were conducted with R version 3.3.2.

## Results

### Characteristics of the study participants

Demographic and clinical characteristics of the nine study subjects are provided in Table 1. One subject was recruited from UMMC and the remaining eight subjects were recruited from MVAMC. Ages ranged from 54 to 82, with a mean age of 72.3. Two subjects were female. Three subjects had mild COPD by GOLD criteria [22] and the remaining six subjects had moderate COPD. Four subjects were active smokers at the time of lobectomy. Subjects had an average of 47.9 pack-years of tobacco exposure. Three subjects were using inhaled corticosteroids.

**Table 1:** Subject characteristics

Subject	Age	Gender	FEV_1_ (% predicted)	COPD severity	Lobectomy site	Current tobacco use	Years since last tobacco use	Total pack-years	ICS^a^ use	LABA^b^ use
1	82	Male	1.28 (64.7)	Moderate	Left upper lobe	No	22	NA^c^	Yes	No
2	77	Male	1.65 (63.1)	Moderate	Right upper lobe	No	0.057	100	No	No
3	69	Male	2.55 (71.8)	Moderate	Right upper lobe	Yes	0	40	No	No
4	73	Male	2.04 (88.9)	Mild	Right middle lobe	No	20	30	Yes	No
5	79	Male	1.99 (78.5)	Moderate	Left upper lobe	Yes	0	40	Yes	No
6	79	Male	2.03 (69.8)	Moderate	Right upper lobe	No	30	45	No	No
7	54	Male	3.55 (90.3)	Mild	Right upper lobe	Yes	0	80	No	No
8	66	Female	2.01 (90.9)	Mild	Left upper lobe	Yes	0	18	No	No
9	72	Female	2.44 (67.1)	Moderate	Right lower lobe	No	12	30	No	No

^a^*ICS* inhaled corticosteroid

^b^*LABA* long-acting beta-agonist

^c^*NA* not available

### More sequences were obtained from subject samples than control samples

Overall, 36 samples from 9 subjects yielded 12,667,009 sequences after quality control, filtering, and OTU clustering steps (Table 2). The mean and median number of sequences per sample was 351,861 and 18,739, respectively, with significantly more sequences obtained from the higher biomass upper airway sites than the lower biomass bronchial and peripheral lung sites. The identical processing of six reagent and laboratory contamination controls yielded 75–820 sequences (mean and median for negative control samples were 113 and 226, respectively). Subsampling down to 563 sequences per sample [32] necessitated the removal of one peripheral lung sample (subject 8) from analysis due to low yield. Only one of six negative control samples yielded greater than 563 sequences.

**Table 2:** Sequencing yield

Subject	Site	Raw sequences	Trimmed^a^	Full data set sequences^b^
1	Oral	1,221,461	1,200,471	1,179,919
1	Nasal	894,311	875,853	730,412
1	Bronchial	720,011	697,567	8380
1	Lung tissue	1,995,525	1,917,602	5978
2	Oral	1,756,838	1,718,429	1,682,345
2	Nasal	940,535	916,270	26,571
2	Bronchial	2,268,600	2,130,104	2811
2	Lung tissue	1,965,320	1,849,877	4270
3	Oral	2,011,911	1,973,158	1,907,839
3	Nasal	1,181,978	1,155,811	818,142
3	Bronchial	1,649,122	1,599,897	10,906
3	Lung tissue	1,649,943	1,573,465	4104
4	Oral	720,179	709,279	697,399
4	Nasal	825,382	795,668	342,890
4	Bronchial	1,031,715	404,201	1050
4	Lung tissue	1,065,648	113,879	1068
5	Oral	593,186	581,011	574,101
5	Nasal	602,148	591,781	562,469
5	Bronchial	997,054	232,540	582
5	Lung tissue	716,643	83,992	684
6	Oral	483,289	470,953	463,259
6	Nasal	619,329	365,827	253,711
6	Bronchial	945,424	9583	691
6	Lung tissue	828,782	18,768	563
7	Oral	899,992	885,538	868,523
7	Nasal	624,109	571,341	378,236
7	Bronchial	671,694	56,354	859
7	Lung tissue	781,672	39,506	931
8	Oral	658,356	646,638	638,699
8	Nasal	582,451	451,154	439,653
8	Bronchial	1,063,865	297,224	1103
*8*	*Lung tissue*	*10,670*	*1307*	*35* ^c^
9	Oral	808,428	798,595	789,718
9	Nasal	1,054,717	304,938	264,946
9	Bronchial	1,286,746	275,686	2161
9	Lung tissue	653,161	87,316	1998
Control	1	19,266	274	115
Control	2	79,459	1956	820
Control	3	22,596	232	103
Control	4	24,344	188	75
Control	5	21,875	348	134
Control	6	23,358	254	111
			Total count^d^	12,667,009
			Median^d^	18,739
			Mean^d^	351,861

^a^Number of sequences that passed trimming steps

^b^Number of sequences that passed trimming, OTU clustering, singleton removal, and removal of taxa not assigned to the 16S rRNA gene. Corresponds to the “Full data set” as noted in Fig. 1

^c^Sample 8-lung tissue was omitted from the analysis due to poor sequencing yield

^d^For samples only, not including control samples

### More 16S rRNA gene copies were observed in upper airway samples than in lower airway samples

**Fig. 2:**
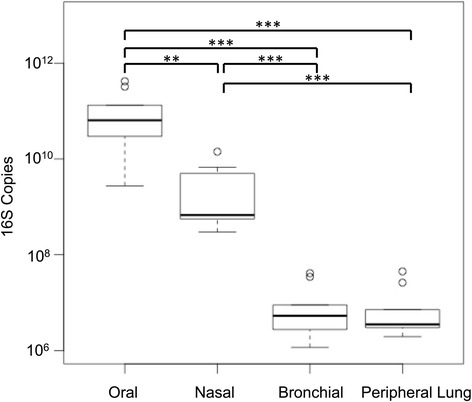
Bronchial and lung tissue microbiota contain two- to fourfold fewer 16S rRNA gene copies than oral and nasal microbiota. Results of 16S rRNA gene qPCR for each sample were determined. 16S rRNA gene copy data were grouped by site. Bronchial and lung tissue microbiota 16S rRNA gene copy numbers were similar. The generalized estimating equations demonstrated an overall *p* < 0.001. Paired *t* tests with Holm correction demonstrated that all pairwise tests were significant with the exception of the bronchial-peripheral lung comparison. The oral-nasal comparison resulted in *p* = 0.004 (**), with the remaining significant comparisons demonstrating *p* values of < 0.001(***)

16S rRNA gene sequencing yields were consistent with the lower 16S rRNA quantitative PCR (qPCR) copy numbers found at the bronchial and peripheral lung sites. qPCR showed that oral communities contained a larger number of 16S rRNA gene copies (1.3 × 10^11^ copies/sample) than nasal communities (3.3 × 10^9^ copies/sample), bronchial communities (1.2 × 10^7^ copies/sample), and peripheral lung communities (1.1 × 10^7^ copies/sample). Methods and results of qPCR studies are available in Fig. 2.

### Alpha diversity is lower in nasal samples than in oral and lung samples

**Fig. 3:**
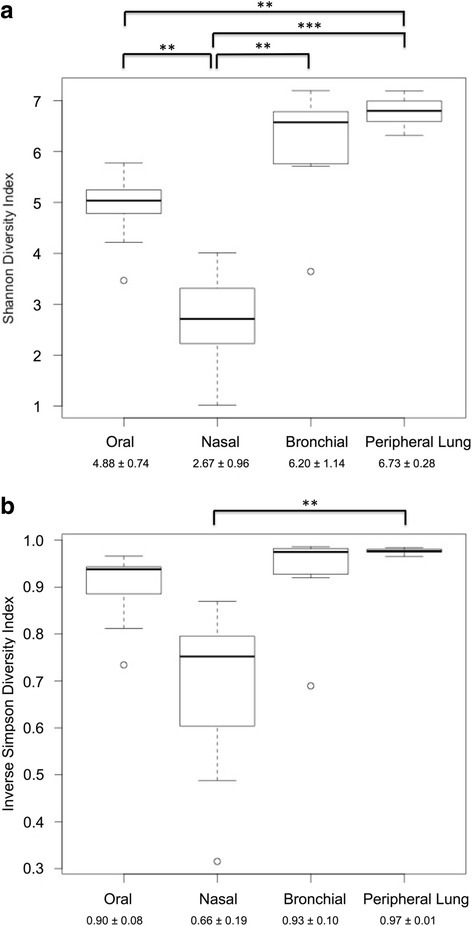
The nasal microbiota is less diverse than the peripheral lung microbiota. The Shannon index (**a**) and Inverse Simpson index (**b**) were used to assess the alpha diversity of each sample. Each box-and-whiskers plot represents one of the anatomic sites (oral, nasal, bronchial, or lung). The dark horizontal bar represents the median value at each site, while the boxes represent the 25th and 75th percentile values. The whiskers indicate the most extreme (non-outlier) data point that is no more than 1.5 times the interquartile range. Outliers are indicated by circles; Three asterisks indicate *p* < 0.001 and two asterisks indicate *p* = 0.01–0.001. The mean ± standard deviation for each site is provided at bottom. Analysis of the Shannon index data (**a**) indicates that diversity is not identical across groups (Generalized Estimating Equations with a batch effect covariate, *p* < 0.001). Post hoc comparison using the Holm correction shows that the nasal microbiota is significantly less diverse than all other sites. The oral microbiota is also significantly less diverse than the peripheral lung microbiota. Analysis of the Inverse Simpson index data (**b**) shows that diversity is not identical across groups (Generalized Estimating Equations with a batch effect covariate, *p* < 0.001). Post hoc comparison using the Holm correction shows that the nasal microbiota is less diverse than the peripheral lung microbiota (*p* = 0.02)

QIIME was used to determine the alpha (within-sample) diversity for each sample. The Shannon diversity index shows that nasal samples are less diverse than oral, bronchial, and lung tissue samples, while oral samples were also less diverse than lung tissue samples (Fig. 3a. Generalized Estimating Equations with batch effect correction, *p* < 0.001 with post hoc pairwise *t* testing accomplished using Holm correction). The inverse Simpson diversity index also shows that nasal samples are less diverse than peripheral lung samples (Fig. 3b, Generalized Estimating Equations with batch effect correction, *p* < 0.001 with post hoc pairwise *t* testing accomplished using Holm correction). The inverse Simpson index did not demonstrate a significant difference in the other pairwise tests. This is likely because the Simpson index, in comparison to the Shannon index, is less sensitive to rare species, which are more prevalent in the bronchial and peripheral lung samples.

### The oral and nasal samples contain distinct microbiota; bronchial and peripheral lung microbiota are a mix of oral and nasal microbiota

**Fig. 4:**
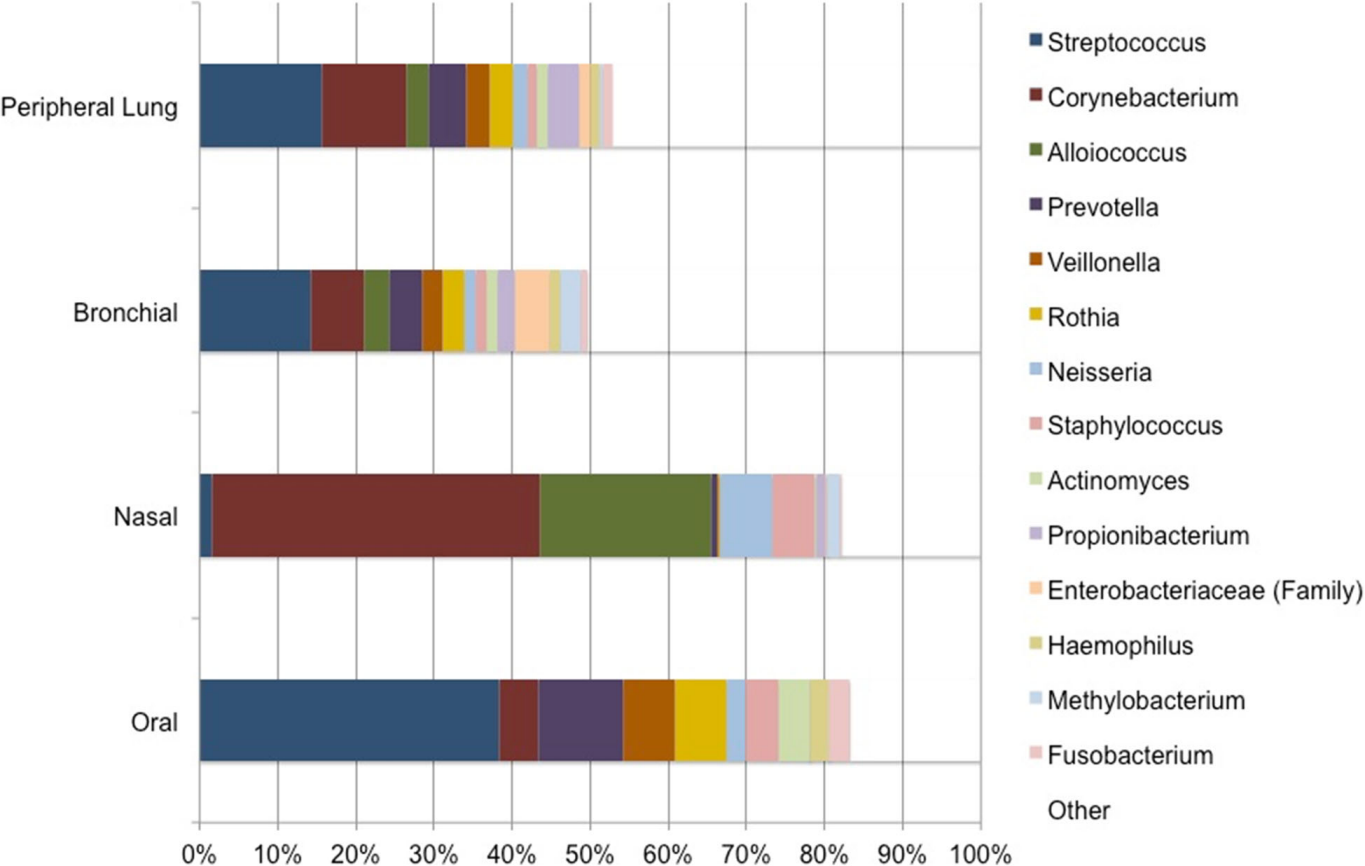
Bronchial and peripheral lung microbiota taxa are similar. Taxonomic assignments for all sequences were determined using Greengenes version 13_8. Each site is represented by a bar, with color indicating genus-level taxonomic assignment and length indicating relative abundance. Taxa present at < 1% overall relative abundance are represented in white. *Streptococcus* (blue) was the most common genus overall (representing nearly 19% of all DNA sequences identified), and the most common genus in the oral samples, bronchial samples, and peripheral lung samples (at 38, 14, and 14%, respectively). In contrast, the nasal samples contained 1.6% Streptococcal sequences. Nasal samples were dominated by *Corynebacterium* (red, 42% of sequences), which was found as 5% of oral sequences. *Corynebacterium* represented 6.6 and 9.4% of bronchial and peripheral lung sequences, respectively

Taxonomic classification of sequences was accomplished using Greengenes version 13_8. Genus-level assignments for all subjects were pooled by site (Fig. 4). Across all 35 samples, the most common genera (in decreasing order) were *Streptococcus*, *Corynebacterium*, *Alloiococcus*, *Prevotella*, *Veillonella*, *Rothia*, *Neisseria*, and *Staphylococcus*. Although all these genera were observed in the bronchial and peripheral lung samples, most of the genera were found predominantly in either the oral or the nasal samples. Oral samples consisted predominantly of *Streptococcus* and also contained *Prevotella*, *Veillonella*, and *Rothia*—but contained few sequences from *Corynebacterium* or *Alloiococcus*. These latter two genera were well represented in the nasal samples, which contained few sequences from *Streptococcus*, *Prevotella*, *Rothia*, or *Veillonella.*

**Fig. 5:**
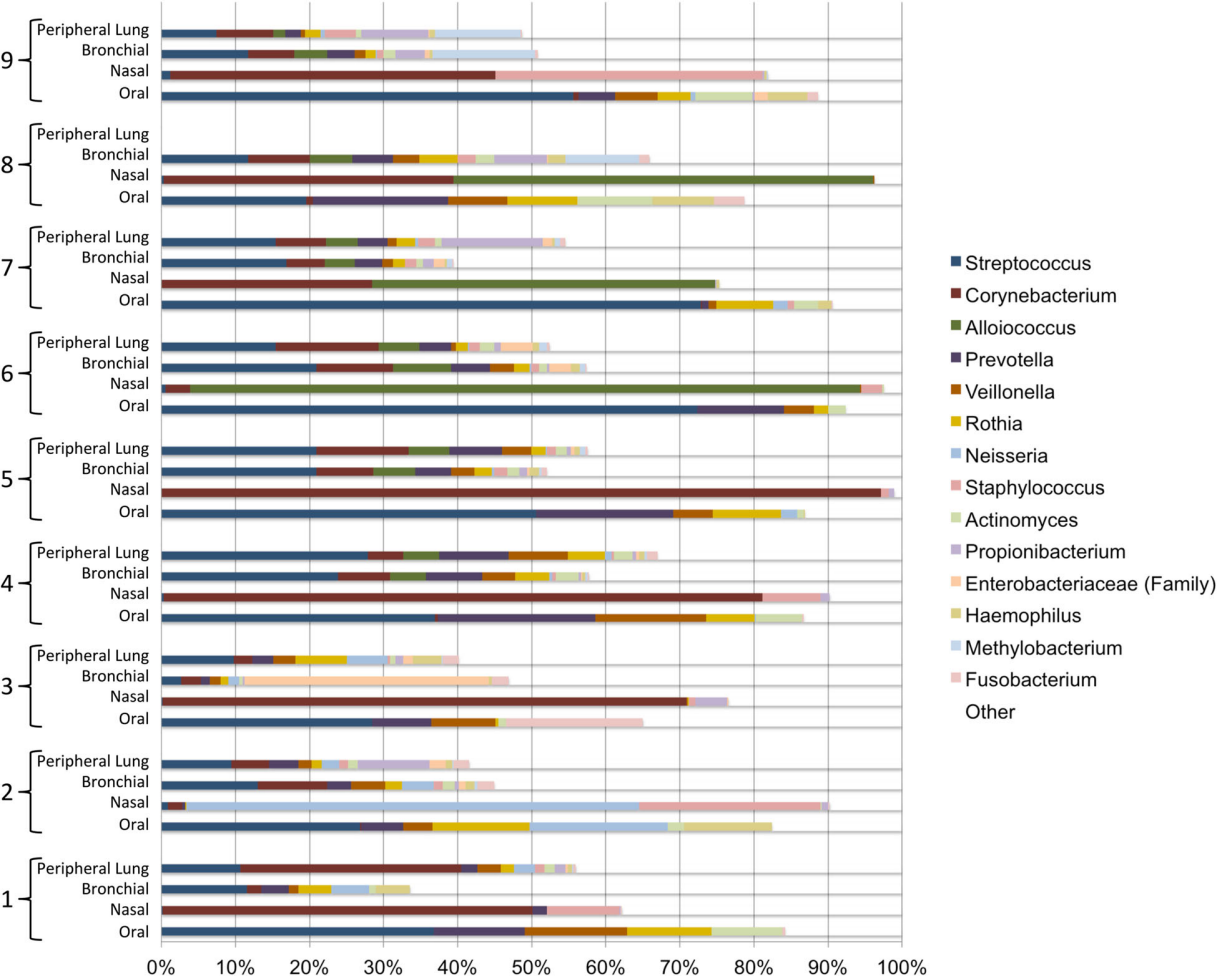
The within-subject bronchial and peripheral lung microbiota are similar. Taxonomic assignments for all sequences were determined using Greengenes version 13_8, and the relative abundance of each genus is provided by both subject and anatomic site. Each bar represents an individual sample, with color indicating genus-level taxonomic assignment and length indicating relative abundance. White was used to represent taxa with < 1% overall relative abundance. Samples are grouped by subject, with oral samples at bottom, then nasal samples, then bronchial samples, and peripheral lung samples at top. Color assignments are indicated in the legend. Each subject’s lung tissue and bronchial microbiota are similar to each other

*Streptococcus* was not only the most abundant genus overall, but also the most abundant genus in the oral, bronchial, and peripheral lung microbiota. Examination of the genus-level taxonomic assignments by subject and site demonstrates that each subject’s bronchial and lung tissue microbiota are very similar to each other (Fig. 5).

### Beta diversity analyses demonstrate within-subject similarity between the bronchial and peripheral lung microbiota

**Fig. 6:**
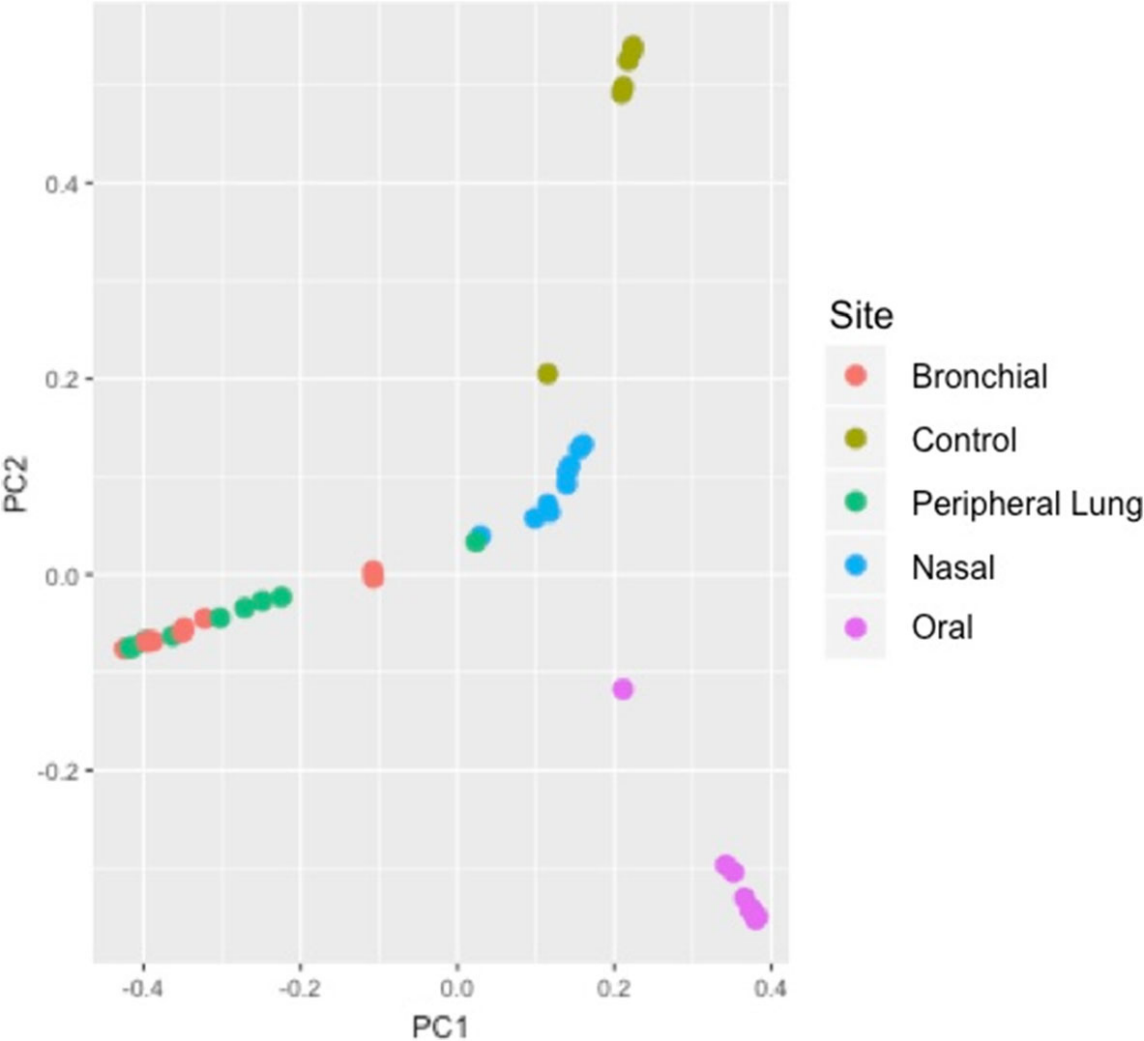
Principal coordinate analysis including negative controls does not demonstrate significant contamination of low-biomass lung-source samples. Principal coordinate analysis with Bray-Curtis distance was performed for all samples and negative controls (extraction and sequencing controls). Negative control samples are indicated in brown, while oral samples are in purple, nasal samples are in blue, bronchial samples are in red, and lung tissue samples are in green. Most negative control samples cluster separately from the clinical samples. One negative control sample appears near the high-biomass nasal samples. The low biomass samples (bronchial and lung tissue samples) do not cluster near the negative control samples

To represent between-sample similarities, β-diversity was assessed using Bray-Curtis similarity. We first assessed the similarity between the reagent and laboratory equipment controls and subject samples prior to normalization and elimination of low sequencing yield samples (Fig. 6, Table 2). Five of the six negative control samples (light brown) formed a distinct cluster in the upper right. The sixth negative control sample (Control 2) was nearby, adjacent to the cluster of high biomass nasal samples (blue). The negative control samples did not cluster near the low biomass bronchial (red) or peripheral lung (green) samples. As background contamination is a much more significant issue in low biomass samples (such as bronchus and lung) than high biomass samples, we concluded that our low biomass bronchial and peripheral lung samples were not heavily influenced by reagent or laboratory equipment contamination. *Lactobacillus* was removed from the full data set prior to rarefaction because it was the only taxon present in the negative control samples and present at > 1% relative abundance in the subject samples.

**Fig. 7:**
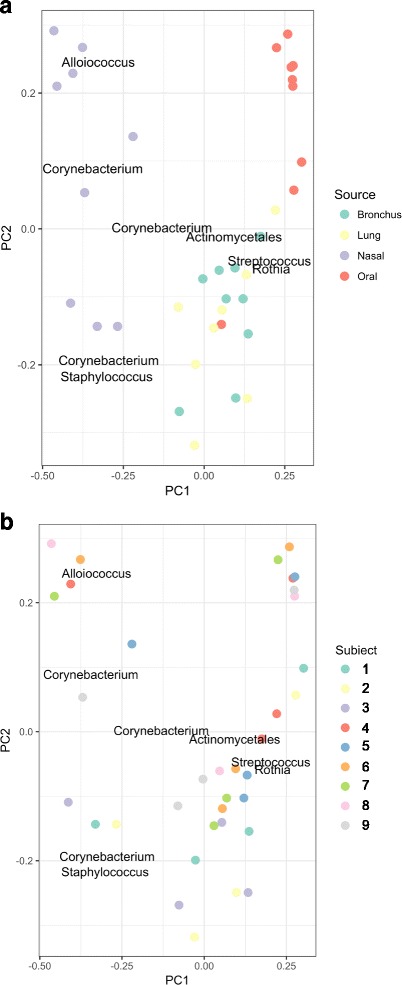
Principal coordinate analysis demonstrates clustering by anatomic site. PCoA of the β-diversity data set using Bray-Curtis distance was performed using QIIME and R following batch-effect correction. (**a**) Oral samples (red) cluster at upper right while nasal samples (purple) are found on the left. Bronchial and peripheral lung samples cluster together in the lower right of the figure. *Alloiococcus*, *Corynebacterium*, and *Staphylococcus* are associated with nasal samples while Actinomycetales, *Streptococcus*, and *Rothia* are associated with bronchial and peripheral lung samples. When all 35 samples are analyzed, clustering by anatomic site is observed (PERMANOVA *p* = 0.001; *r*^2^ = 0.275; heterogeneous dispersion). (**b**) Samples were color-coded by subject (see legend). When all 35 samples were included in the analysis, there was no clustering by subject (PERMANOVA *p* = 0.44; *r*^2^ = 0.238; homogeneous dispersion)

We then proceeded with rarefaction of subject samples at 563 sequences. Preliminary PERMANOVA analyses demonstrated a sequencing batch effect, so a linear model was applied to the rarefied data set to account for batch effects, which resulted in the β-diversity data set. PCoA using Bray-Curtis distance with all 35 samples labeled by anatomic site demonstrates clustering by anatomic site Fig. 7a). Oral samples (red) cluster in the upper right, while nasal samples (purple) are found on the left. Bronchial and peripheral lung samples (green and yellow, respectively) cluster together in the lower right of the plot. As expected, *Alloiococcus*, *Staphylococcus*, and *Corynebacterium* were associated with nasal samples, while *Streptococcus*, Actinomycetales, and *Rothia* were associated with bronchial and peripheral lung samples. PERMANOVA demonstrated clustering by anatomic source (*p* = 0.001, *r*^2^ = 0.275; heterogeneous dispersion). PCoA and PERMANOVA do not demonstrate clustering by subject (Fig. 7b, *p* = 0.44, *r*^2^ = 0.238; homogeneous dispersion).

**Fig. 8:**
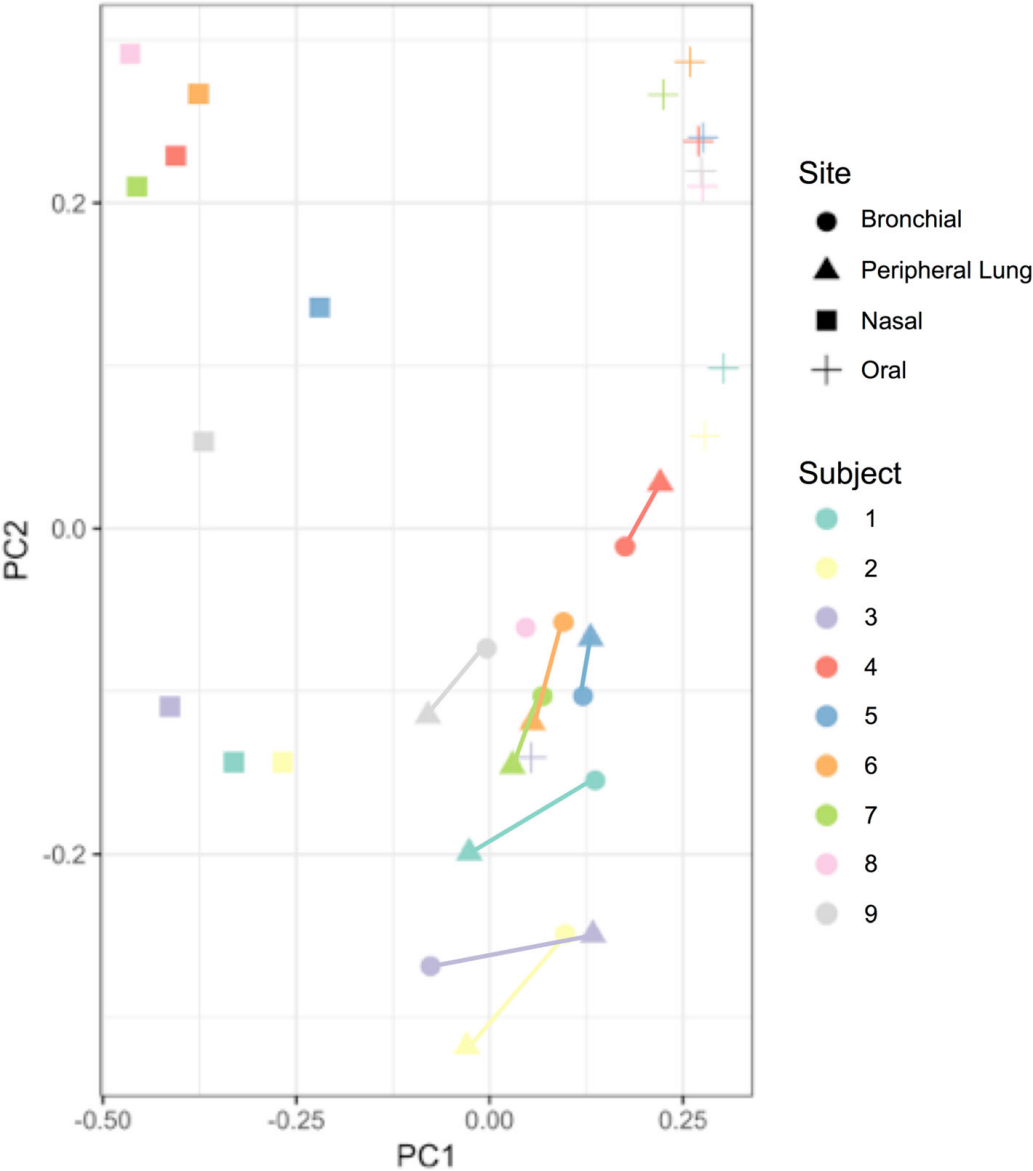
Principal Coordinate Analysis of All Subject Samples Does Not Demonstrate Clustering by Subject. PCoA of the β-diversity data set using Bray-Curtis distance was performed using QIIME and R. Samples were color-coded by subject with the sample site noted by shape (see legend). Bronchial (circles) and peripheral lung samples (squares) from the same subject are connected with colored subject-specific lines. In an analysis of the 17 bronchial and peripheral lung samples only, the within-subject distance between the bronchial and peripheral lung microbiota is smaller than the between-subject distance of the bronchial and peripheral lung microbiota (permutation testing with batch correction, *p* = 0.0139)

When the same analysis was replotted to indicate both anatomic site and subject, the similarities between the bronchial and peripheral lung microbiota are even more clear (Fig. 8). Anatomic site is now indicated by symbol shape, while subject number is indicated by color. Like-colored lines are included to represent the distance between each subject’s own bronchial and peripheral lung microbiota. Each subject’s peripheral lung microbiota is found in close proximity to his/her own bronchial microbiota. The within-subject distance between the bronchial and peripheral lung microbiota is smaller than the between-subject distance between the bronchial and peripheral lung tissue microbiota (permutation test with batch correction, *p* = 0.0139). This shows that the two lung samples from the same subject are more similar to each other than are two lung samples from two different subjects.

### The lung microbiota contains more contributions from the oral microbiota than from the nasal microbiota

To determine the relative contributions of the upper airway (oral and nasal) microbiota to the lower airway (bronchial and lung tissue) microbiota, we used SourceTracker. SourceTracker identified that the lung tissue microbiota reflects 21.1% oral source, 8.7% nasal source, and 70.1% unknown source. The bronchial microbiota reflects 22.7% oral source, 5.5% nasal source, and 71.7% unknown source (all percentages are the means across all subjects, Table 3).

**Table 3:** Relative contributions of oral and nasal microbiota to the lung microbiota

Subject and site	Oral (% ± SD^a^)	Nasal (% ± SD^a^)	Unknown (% ± SD^a^)
1 Bronchus	16.9 ± 0.62	1.3 ± 0.23	81.6 ± 0.71
2 Bronchus	22.0 ± 0.60	4.0 ± 2.1	74.0 ± 2.4
3 Bronchus	5.7 ± 0.49	1.2 ± 0.64	93.1 ± 0.70
4 Bronchus	42.9 ± 0.62	4.7 ± 0.30	52.4 ± 0.76
5 Bronchus	25.9 ± 0.97	5.4 ± 0.36	68.7 ± 0.99
6 Bronchus	27.9 ± 0.34	12.0 ± 0.27	60.0 ± 0.36
7 Bronchus	17.9 ± 0.36	8.0 ± 0.32	74.0 ± 0.42
8 Bronchus	29.9 ± 0.32	11.1 ± 0.35	59.1 ± 0.46
9 Bronchus	15.4 ± 0.78	1.9 ± 0.25	82.6 ± 0.84
Bronchus average	22.7%	5.5%	71.7%
1 Peripheral lung	16.9 ± 0.70	13.5 ± 1.05	69.8 ± 1.1
2 Peripheral lung	8.8 ± 0.75	13.3 ± 2.9	77.9 ± 3.4
3 Peripheral lung	13.5 ± 1.0	1.6 ± 0.57	84.9 ± 1.4
4 Peripheral lung	53.9 ± 0.71	3.2 ± 0.27	42.9 ± 0.70
5 Peripheral lung	30.4 ± 0.33	10.5 ± 0.37	59.1 ± 0.39
6 Peripheral lung	18.2 ± 0.57	7.7 ± 0.35	74.1 ± 0.78
7 Peripheral lung	16.9 ± 0.66	11.0 ± 0.84	72.1 ± 1.2
8 Peripheral lung	NA^b^	NA^b^	NA^b^
9 Peripheral lung	9.9 ± 0.45	9.1 ± 0.77	80.1 ± 0.58
Peripheral lung average	21.1%	8.7%	70.1%

^a^Standard deviation

^b^Not applicable, due to low sequencing yield

### The neutral theory of community ecology can describe the peripheral lung microbiota using the upper airway microbiota

**Fig. 9:**
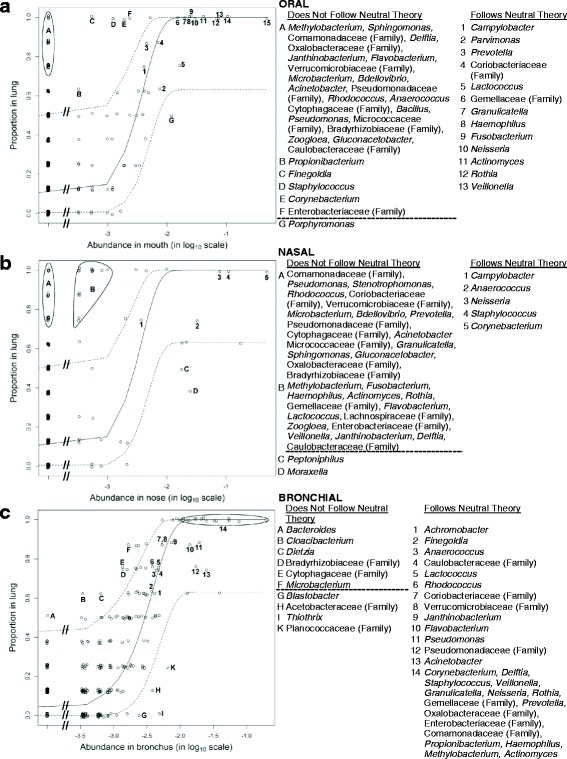
The neutral theory of community ecology demonstrates that the bronchial and lung tissue microbiota are very closely related. Neutral model comparing the source microbiota with the lung tissue microbiota. Each figure contains a solid line that represents the expected proportion of an OTU in the lung at a given source abundance. The dotted lines represent the 95% confidence interval of the line. The *x*-axis is presented in log scale for clarity and ease of presentation. Taxa found in the lung but not in the source site are included on the far left of each figure, and a truncated *x*-axis break is used to indicate that these data points are displayed despite the use of the log scale. (**a**) When the oral microbiota was used as the source community for the lung tissue microbiota, OTUs corresponding to common oral taxa (labeled 1–15; e.g., *Streptococcus*, *Prevotella*, *Veillonella*, *Actinomyces*) follow the neutral theory. Many OTUs not found in the oral site (labeled A) and low abundance oral OTUs (labeled B) were more common in the lung tissue than would be predicted by the neutral theory. One common oral OTU (*Porphyromonas*, labeled G) was less common in the lung tissue than was predicted by the neutral theory. The immigration probability for oral taxa was 0.62. (**b**) When the nasal microbiota was used as the source community for the lung tissue microbiota, OTUs corresponding to common nasal taxa (labeled 1–5; e.g., *Corynebacterium*, *Staphylococcus*, *Neisseria*) follow the neutral theory. Many OTUs not found in the nasal site (labeled A) and low abundance nasal OTUs (labeled B) were more common in the lung tissue than would be predicted by the neutral theory. Notably, many of these low nasal abundance OTUs are common oral taxa (e.g., *Fusobacterium*, *Actinomyces*, *Prevotella*, *Veillonella*). Two common nasal abundance OTUs (labeled C and D, including the common COPD pathogen *Moraxella*) were less common in the lung tissue than was predicted by the neutral theory. The immigration probability for nasal taxa was 0.74. (**c**) When the bronchial microbiota was used as the source community for the lung tissue microbiota, OTUs corresponding to common bronchial taxa (labeled 1–14, including both common oral and nasal taxa such as *Streptococcus*, *Corynebacterium*, *Prevotella*, and *Veillonella*) follow the neutral theory. Several low abundance bronchial OTUs (labeled A–F) were more common in the lung tissue than was predicted by the neutral theory. In contrast to the oral and nasal source figures, only one OTU (labeled A, *Bacteroides*) was not found in the bronchus but was more common in the lung tissue than would be predicted by the neutral theory. Three moderate bronchial abundance OTUs (labeled G–K) were less common in lung tissue than was predicted by the neutral theory. The bronchial microbiota immigration probability was 0.69

We utilized the neutral theory of community ecology to identify OTUs at independent source sites (oral, nasal, or bronchus) that do or do not predict that same OTU’s presence in the lung tissue. Using only the oral microbiota as the source site, many oral taxa (*Streptococcus*, *Prevotella*, *Veillonella*, *Rothia*, *Actinomyces*, *Neisseria*, *Fusobacterium*, *Haemophilus*, and *Prevotella*) appear in the lung tissue microbiota consistent with the neutral theory (Fig. 9a). The abundance of one oral taxon (*Porphyromonas*) and three nasal-associated taxa (*Corynebacterium*, *Staphylococcus*, and *Propionibacterium*) does not appear consistent with the neutral theory of community ecology. The immigration probability for the oral microbiota is 0.62, indicating that for each microbe that dies in the lung, there is a 62% chance that it will be replaced by a microbe from the oral compartment and a 38% chance that it will be replaced by a microbe from the lung. Using only the nasal microbiota as the source site, several nasal taxa (*Corynebacterium*, *Staphylococcus*, and *Neisseria*) appear in the lung tissue microbiota consistent with the neutral theory (Fig. 9b). One known member of the upper respiratory tract microbiota and common COPD pathogen, *Moraxella*, was not consistent with the neutral theory and found less frequently in the lung tissue microbiota than its abundance in the nasal microbiota predicted. Most upper airway taxa are found in either the mouth or the nose, but not in both locations. Not unexpectedly, the nasal abundance of many oral-associated taxa (e.g., *Veillonella*, *Fusobacterium*, *Haemophilus*, *Actinomyces*, *Rothia*, and *Prevotella*) was not predictive of the lung tissue prevalence using the neutral theory. The nasal microbiota immigration probability was 0.74.

Using the bronchial microbiota as the only source for the lung tissue microbiota, 11 of the 14 most common taxa in our data set were consistent with the neutral theory (Fig. 9c). The immigration probability for the bronchial microbiota was 0.69. Taxa not consistent with the neutral theory using the bronchus as the source community were neither common in our data set nor common lung pathogens.

## Discussion

This is the first study to empirically determine the lung tissue microbiota in mild to moderate COPD patients without passing the lung samples through the oropharynx. Our results demonstrate that the oral taxa identified in prior studies of the COPD lung microbiota are due to a physiologic process such as aspiration, rather than contamination of samples during bronchoscopy or expectoration. Our work confirms the work of Dickson et al., which used bronchoalveolar lavage to provide evidence that bacteria enter the lungs primarily through microaspiration [13]. The COPD bronchial and lung tissue microbiota are very similar and consist of *Streptococcus*, *Corynebacterium*, *Alloiococcus*, *Prevotella*, *Veillonella*, and *Rothia*. We found that the upper airway microbiota may be predictive of the lung tissue microbiota. While the data overall provide support for the neutral theory in the COPD lung, there is also evidence of ecologic drift of some clinically relevant taxa (such as *Porphyromonas* and *Moraxella*)—consistent with some selective pressure on bacteria in the COPD lung.

As it is impractical to use operatively obtained tissue samples in future lung microbiota studies, there is a need to determine which non-invasive sampling methods most accurately reflect the lung tissue microbiota. The lung lobectomy protocol utilized here is well suited to determining the best samples and reasonable non-invasive samples to use in future studies of the lung microbiota.

The taxa identified in our nasal and oral samples are very similar to the taxa identified in earlier studies [2, 33–36], but we found very little overlap between oral and nasal communities. Bronchial and lung tissue samples reflected a mix of the two source communities. This is in contrast to some earlier work [3, 4, 13], which identified fewer nasal-associated taxa (such as *Corynebacterium* or *Propionibacterium*) in BAL samples from the lung than were identified in our tissue samples. We did not observe *Tropheryma* in any sample and therefore cannot address previous studies suggesting that *Tropheryma whipplei* is disproportionately abundant in the lung relative to the upper respiratory tract [9]. The Streptococci are a large genus containing numerous species and serotypes that are adapted to colonization or infection of diverse human sites (i.e., oropharynx, lung, skin). Our study was unable to differentiate between oral Streptococci and *Streptococcus pneumoniae*, a common COPD lung pathogen and cause of pneumonia, because 16S rRNA gene hypervariable region 3 sequences are of insufficient length to discriminate between various species in the genus *Streptococcus*. Furthermore, our study identified relatively few *Haemophilus* or *Moraxella* in the lung, both of which are typically associated with exacerbations or airway colonization in COPD. This is likely because our study subjects represented a less severe COPD phenotype and were unlikely to experience frequent exacerbations. Although we did not structure our inclusion/exclusion criteria to select for a less severe COPD phenotype, our subjects were limited to those whose lung function and co-morbidities did not preclude lung lobectomy.

Two prior studies have analyzed the lung tissue microbiota of end-stage COPD at the time of lung transplantation [3, 21]. These studies identified different microbiota present at different sites within the same subject. These findings suggest that the lung site (upper lobe, lower lobe, etc.) in addition to anatomic and physiologic changes in the lung as a result of COPD progression and treatments may alter the lung microbiota. For clinical reasons, our study was limited to evaluating only one lung lobe per subject. Therefore, we cannot evaluate potential alterations in the lung microbiota based on anatomic site. Additionally, Kitsios et al. [37] recently described the lung tissue microbiota of end-stage idiopathic pulmonary fibrosis (IPF) and healthy donor lung unsuitable for transplant. They found that IPF lung tissue contained typical skin microbiota (Comamonadaceae and *Methylobacterium*) and was indistinguishable from background signal. In contrast, their healthy donor lung tissue contained *Streptococcus* and *Prevotella*. Our lung samples were much more similar to the healthy donor lungs rather than the IPF lungs and background contamination studied by Kitsios et al. *Streptococcus* and *Prevotella* were the 1st and 4th-most abundant genera in our lung samples, respectively, while skin organisms/background contaminants were less common in our lung samples (*Methylobacterium* was the 13th most abundant taxa in our data set; we did not observe Comamonadaceae). *Corynebacterium*, a common skin organism, was the second most common taxa in our dataset. However, it was preferentially observed in the nasal samples, likely due to the close proximity between skin and nose.

Our study of the COPD lung tissue microbiota identified several of the same bacteria found in previous studies of the COPD lung microbiota using BAL. Of the seven most common bacteria identified in the Erb-Downward et al. study, our study also identified two taxa (*Streptococcus*, *Veillonella*) among our seven most common taxa [3]. Of the six most common bacteria identified by Hilty et al., we also identified three taxa (*Streptococcus*, *Veillonella*, *Neisseria*) [4]. Of the seven genera identified by Cabrera-Rubio et al., we also identified two taxa (*Streptococcus*, *Neisseria*) [38]. Of the taxa found in BAL studies but not found among our most common taxa, only three (*Prevotella*, *Fusobacterium*, and *Haemophilus*) were found in more than one of the BAL-based studies. Notably, these three taxa were also identified in our study, but in lesser relative abundance. We also previously published a study of the lung microbiota in moderate and severe COPD using BAL samples [1]. Despite the difference in sample acquisition techniques (BAL fluid vs. tissue swabs), the lung taxa observed in our two studies were similar. Our BAL-based study identified *Actinomyces*, *Streptococcus*, *Propionibacterium*, *Corynebacterium*, *Devosia*, *Rothia*, and *Haemophilus* as the most abundant genera in the COPD lung microbiota. The present study also identified *Streptococcus*, *Corynebacterium*, and *Rothia* among the seven most abundant taxa in the COPD lung microbiota. Our present lung microbiota findings are similar to our own and others’ previous results obtained using BAL samples.

Our analysis using SourceTracker showed that approximately 30% of a subject’s lower airway microbiota reflects the taxonomic composition and relative abundances of the subject’s upper airway samples. The remaining 70% of the lower airway microbiota was not attributed to an upper airway source using this technique. SourceTracker applies a Bayesian approach to determine the relative contributions of one or more designated “sources” to a particular “sink” microbiota, while modeling the uncertainty regarding known and unknown source environments. The program also assigns a portion of the microbiota to an “unknown” source, which represents several possibilities: contamination introduced by laboratory reagents or equipment, incompletely sequenced source communities, one or more unknown/unsampled sources, and the presence of a dynamic “target” microbiota capable of selecting for the growth or maintenance of certain taxa but not others. There are several factors that likely combined to suggest that the majority of the microbiota are not the direct result of aspiration. One potential explanation is contamination of the lung samples during extraction, amplification, and sequencing. This is always a concern during the molecular analysis of low biomass samples; however, we took several steps to minimize this potential issue. Samples were subjected only to the minimum number of PCR cycles necessary to amplify a product, reagent and environmental controls processed alongside the samples did not amplify a PCR product, control samples did not appear similar to low biomass samples on multidimensional scaling (Fig. 6), and the most common control taxa (*Lactobacillus*) was removed from the data set prior to further analysis. Furthermore, there was a statistically significantly smaller distance between each subject’s paired bronchial and lung tissue samples than the between-subject distance at these two sites in a permutation analysis, which would not be expected if contamination of these low biomass samples was one of the primary factors responsible for these observations. A second potential contributor to the “unknown” lung microbiota is an unsampled site. It is possible that an unsampled upper airway niche (i.e., dental plaque) or a part of the environment (i.e., air) contributes to the lung microbiota. A third potential explanation is incomplete sampling of one of the known source sites. This is unlikely, as the rarefaction curves for the oral and nasal samples indicate thorough sampling of these sites (data not shown). The fourth possibility is the presence of a dynamic microbiota at all sites so that the lung microbiota at a given time point does not simply reflect the content and relative abundance of the source communities at the same time. It is possible that “ecologic drift” occurs, allowing some taxa to grow in the lung, while others are selectively removed by the immune system or mucociliary clearance.

The neutral theory of community ecology attempts to predict community composition based on the known composition of a relevant source community. In this theory, community composition is not influenced by any organism’s inherent biological suitability for the source environment compared to the new environment. Therefore, OTUs that follow the neutral theory can be predicted based on the source community composition. OTUs that do not follow the neutral theory may potentially indicate ecologic drift of that organism in the new target environment. Our neutral theory of community ecology studies identified the oral and nasal microbiota as important sources of the lung tissue microbiota, with a 62 or 74% probability, respectively, that an OTU that dies in the lung tissue will be replaced by an organism from one of these sources. Conversely, there is a 38 or 26% probability that ecologic drift will occur (replacement of the dead OTU with a lung tissue OTU), rather than replacement with an oral or nasal OTU, respectively. Given the close anatomic proximity between the bronchus and the lung tissue, it is therefore not surprising that the neutral theory also holds very well for the association between the bronchial and lung tissue microbiota, with a 69% probability that a lung tissue OTU which dies will be replaced by an OTU from the bronchus. We note that the statistical techniques used to calculate the immigration probability are unable to reliably calculate a confidence interval due to the small sample size, so we are unable to conclude which source or sources most contribute to the lung microbiota.

Venkataraman et al. published a study of the healthy lung microbiota and the cystic fibrosis (CF) and idiopathic interstitial pneumonia (IIP) lung microbiota. They applied the neutral community model and found that the healthy lung microbiota is consistent with aspiration from the oral cavity with little or no selection of taxa in the healthy lung (the adapted island model). In contrast, the CF and IIP lung microbiota selected for certain taxa [39]. Dickson et al. compared the upper airway and lung microbiota of healthy subjects using BAL and determined that the similarities between the upper airway and lung microbiota increased when the lung sample was obtained from a more proximal lung site. This study also supports the adapted island model [12]. Bassis et al., in their study of healthy lung using BAL samples, concluded that the healthy lung is able to selectively eliminate *Prevotella* aspirated from the oropharynx [14]. Our study suggests that *Porphyromonas* or *Moraxella* may be selectively eliminated from the early-stage COPD lung tissue microbiota. It is possible that lungs with more severe COPD or more frequent exacerbations may exhibit more ecologic drift.

Our studies using SourceTracker suggested that the lung tissue microbiota exhibited significant ecological drift, beyond what was shown using the neutral theory. These two analyses are modeling similar but distinct phenomena: SourceTracker models the composition of the microbiota at one time point, while the neutral theory models replacement of “dead” microbiota over time. Taken together, the two different analyses indicate that while the lung microbiota is frequently replaced by nasal and oral taxa, over time the composition of the lung microbiota bears less and less resemblance to the upper airway sources. Another important distinction between the models is that SourceTracker considers multiple sources simultaneously, while the neutral theory only considers one source at a time. SourceTracker also appears to be more sensitive to the many low-abundance taxa found in the lung and bronchus.

Despite the limitations of our study (small sample size, lack of non-COPD control subjects, and the inability to discriminate between oral Streptococcal species and *S. pneumoniae*), we have demonstrated that oral taxa are present in the lungs of subjects with COPD due to a physiologic process such as aspiration, rather than due to sample contamination at the time of acquisition. Our findings in mild and moderate COPD may not be representative of the healthy lung microbiota or the lung microbiota of severe or very severe COPD.

Future work using this lung lobectomy protocol should be undertaken to improve our lung tissue microbiota predictive capability and examine the use of additional non-invasive surrogate samples to model the COPD lung microbiota. Increased sampling intensity of the oropharynx and lung tissue sites will ensure that all sites are exhaustively sequenced and provide information on the reproducibility and stability of these microbiota. Inclusion of induced sputum samples from these subjects prior to lobectomy will allow us to evaluate the agreement between lung tissue microbiota, the bronchial microbiota, and induced sputum microbiota.

## Conclusions

Using a technique that avoids oral contamination of the lung sample, we found that the mild or moderate COPD lung tissue microbiota contains upper airway taxa. Our study is significant because it is the first study to empirically demonstrate that the oral bacteria found in the COPD lung are present due to a physiological process, such as aspiration, rather than upper airway contamination during the experimental procedure. The lung sampling technique reported here may be used in future studies to validate non-invasive surrogate samples for studying the COPD lung tissue microbiota.
